# Construction of an m6A- and neutrophil extracellular traps-related lncRNA model to predict hepatocellular carcinoma prognosis and immune landscape

**DOI:** 10.3389/fimmu.2023.1231543

**Published:** 2023-10-05

**Authors:** Tian Zhan, Wei Wang, Xiao Guan, Wei Bao, Na Lu, Jianping Zhang

**Affiliations:** ^1^ Department of General Surgery, The Second Affiliated Hospital of Nanjing Medical University, Nanjing, Jiangsu, China; ^2^ Department of Clinical Laboratory, Lianshui County People’s Hospital, Huai’an, China; ^3^ Department of Urology, The Second Affiliated Hospital of Nanjing Medical University, Nanjing, China

**Keywords:** long non-coding RNA, N6-methyladenosine, neutrophil extracellular traps, hepatocellular carcinoma, bioinformatics

## Abstract

**Purpose:**

To investigate the impact of N6-methyladenosine- (m6A) and neutrophil extracellular traps- (NETs) related lncRNAs (MNlncRNAs) on the prognosis of hepatocellular carcinoma (HCC).

**Methods:**

We collected m6A and NETs-related genes from published studies. We identified the MNlncRNAs by correlation analysis. Cox regression and the least absolute selection operator (LASSO) method were used to select predictive MNlncRNAs. The expressions of predictive MNlncRNAs were detected by cell and tissue experiments. Survival, medication sensitivity, and immunological microenvironment evaluations were used to assess the model’s prognostic utility. Finally, we performed cellular experiments to further validate the model’s prognostic reliability.

**Results:**

We obtained a total of 209 MNlncRNAs. 7 MNlncRNAs comprised the prognostic model, which successfully stratifies HCC patients, with the area under the curve (AUC) ranging from 0.7 to 0.8. *In vitro* tests confirmed that higher risk patients had worse prognosis. Risk score, immunological microenvironment, and immune checkpoint gene expression were all significantly correlated with each other in HCC. In the group at high risk, immunotherapy could be more successful. Cellular assays confirmed that HCC cells with high risk scores have a higher proliferation and invasive capacity.

**Conclusion:**

The MNlncRNAs-related prognostic model aided in determining HCC prognosis, revealing novel therapeutic options, notably immunotherapy.

## 1 Introduction

Liver cancer has a poor prognosis and ranks third in the list of tumor-related causes of death (1). The most frequent kind of liver cancer is hepatocellular carcinoma (HCC) (2). With the development of medicine, great progress has been made in the treatment of HCC (3). Surgery and liver transplantation are currently the common treatment options for HCC (4, 5). However, the prognosis of HCC patients does not increase with the progression of treatment. Five-year survival for patients with advanced HCC is only 10%, with a median survival of fewer than 10 months (6). HCC is a rather complex disease with a high degree of heterogeneity that poses a challenge to the prognostic assessment of patients (7). For immunotherapy of HCC, the identification and validation of predictive biomarkers remain a major unresolved challenge (8).

Long non-coding RNAs (lncRNAs), which are usually not translated into proteins, regulate gene expression involved in cell growth and proliferation (9). According to research, lncRNA is a potential biomarker that plays a critical function in tumor formation (10). Meanwhile, the lncRNA model can reliably predict the outcome of cancer patients and recommend therapeutic therapy (11). Therefore, it is practical to study lncRNA to evaluate the outcome of patients with HCC (12).

N6-methyladenosine (m6A) is the most common RNA modification and a hot issue in the field of cancer research and is abundantly represented in the transcriptome (13, 14). M6A affects almost all aspects of RNA metabolism, providing new ideas for cancer diagnosis, treatment, and prognosis assessment (15). The tumor immune microenvironment is an important element in the growth and spread of malignancies (16). The majority of immune system cells in humans are neutrophils, which serve as tumor patients’ biomarkers for risk classification (17, 18). Especially in HCC, the presence of tumor-associated neutrophils is linked to poor prognosis (19). Neutrophil extracellular traps (NETs) are reticulations whose function is mainly to kill harmful microorganisms (20). Classical NETs are formed by a process known as “NETosis”, which is distinct from programmed cell death and is a certain type of controlled cell death (21). NETs are important for tumor development. By stimulating the immune system, NETs prevent tumor development, whereas tumors can instruct neutrophils to undergo NETosis in order to promote metastasis (22).

Studies had demonstrated that the expression of m6A-related genes was closely related to the immune microenvironment of malignant tumors and can guide patient immunotherapy (23). Meanwhile, m6A genes were mainly enriched in the formation of extracellular traps in neutrophils, suggesting that probing the m6A patterns of tumors can help to understand the diversity and complexity of the tumor microenvironment (23). It has been shown that models that integrate multiple markers into a single model outperform those constructed using a single marker, facilitating individualized patient management (24). Consequently, combining several kinds of biomarkers is acceptable to create a more accurate model.

Hence, we first identified a set of m6A- and NETs-related lncRNAs (MNlncRNAs) linked to m6A genes and NETs-related genes. We developed a predictive model that can reliably predict patients’ survival from HCC based on these MNlncRNAs. Also, the immune microenvironment and drug sensitivity of HCC were correlated with MNlncRNAs. This research lays the groundwork for an HCC treatment plan.

## 2 Materials and methods

### 2.1 Data acquisition

Transcriptome and clinical data were obtained from the TCGA database. Counts was the workflow type utilized. All data were log2 transformed. All the HCC tissues and adjacent tissues were gathered from 20 HCC patients who had received curative surgery at Second Affiliated Hospital of Nanjing Medical University between 2020 and 2021. The utilization of human tissues was granted ethical approval by the ethics committee of the Second Affiliated Hospital of Nanjing Medical University. Meanwhile, we collected 23 m6A-related genes and 69 NETs-related genes from the published studies. 23 m6A genes were m6A regulators, including 13 readers, 8 writers and 2 erasers (25). The NETs-related gene set composed of 69 genes summarized the research progress of NETs in immunity and various diseases, mainly covering the ligands and receptors that stimulate the formation of NETs, downstream-related signals, and the molecules identified to adhere to the framework of NETs (26, 27).

### 2.2 Identification of MNlncRNAs

By Pearson correlation analysis, we identified a set of m6A-related lncRNAs and NETs-related lncRNAs. The p-value was set to 0.001 and the correlation coefficient was set at 0.40. Then, We obtained a set of MNlncRNAs by taking the intersection of these two sets of lncRNAs. These MNlncRNAs were used for subsequent analysis.

### 2.3 TCGA data process

Before obtaining the count file, the downloaded data were combined and preprocessed with the Perl programming language. The lncRNA symbols were changed using Perl. The TCGA transcriptome data were then matched to MNlncRNAs. Patients with insufficient clinical data and no follow-up days were eliminated. We performed differential analysis (p<0.001) of normal and tumor samples to screen out MNlncRNAs with differential, which were matched to the survival data. In addition, to reduce the effect of noise, patients with zero expression of MNlncRNAs were removed, and a 7:3 ratio of training cohorts (218) to test cohorts (98) was generated from the TCGA dataset at random.

### 2.4 Prognostic model construction and evaluation

After matching the MNlncRNAs expression data and clinical data, the univariate COX analysis was performed (p <0.001). Then, the LASSO regression method was utilized to narrow down the list of MNlncRNAs with prognostic significance. The risk score was computed according to the model formula. After that, the prognostic model was built. The prognostic model’s performance was validated using the test cohorts.

We divided them into high- and low-risk categories based on the median. Following that, we ran a survival analysis to check if there was any difference in prognosis between the training and test cohorts. Meanwhile, we displayed the sample distribution between the two cohorts to assess the efficacy of stratifying samples. Heatmaps were utilized to compare the expression levels of the model MNlncRNAs. The AUC was used to validate the model’s prediction capabilities. In addition, we compared the predictive performance of risk scores with clinical factors.

### 2.5 Functional analysis

The Gene Ontology (GO) and Kyoto Encyclopedia of Genes and Genomes (KEGG) pathway studies were performed using the “clusterProfiler” package. To do gene set variation analysis (GSVA), we used the “GSVA” package. The results were shown using bar charts (p<0.05).

### 2.6 Cell culture

HCC cell lines, including PLC, SK, LM3, HepG2 and HuH-7, and human liver cell LO2 were obtained from the American Type Culture Collection (ATCC) and cultured in DMEM medium containing 10% FBS.

### 2.7 Quantitative real-time polymerase chain reaction (qRT-PCR)

Utilizing the TRIzol method (Invitrogen, Carlsbad, CA, USA) to extract total RNA from cells or tissues. Reverse transcription followed the instructions of PrimeScriptTM RT reagent kit (TaKaRa, Kyoto, Japan) and qRT-PCR followed the instructions of the CHAMQ SYBR qPCR Master Mix kit (Vazyme, Nanjing, China). The relative gene expression was calculated using the 2^-△△CT^ method. All primers sequences in our research are listed in **Supplementary Table 1**.

### 2.8 Transwell migration and invasion assay

Utilizing transwell chambers (8 μm PET; Millipore Corporation, Burlington, MA, USA) to perform transwell assay. Cells (2×105/ml) were resuspended in serum-free DMEM medium. For the migration assay, 200µl of the cell suspension was added to the upper chamber, and 800µl of DMEN medium supplemented with 10% serum was added to lower chamber. For the invasion assay, 200µl of the cell suspension was added to the upper chamber precoated with 0.5 mg/L Matrigel (BD Biosciences, Franklin Lakes, NJ, USA), and 800µl medium containing with 10% serum was added to lower chamber. After 24 hours of incubation, cells were fixed with methanol for 30 minutes and stained with 0.4% crystal violet for an hour at room temperature. The upper layers of cells were gently erased with cotton swabs, and the chambers were washed 3 times with PBS. The cells were counted in 5 random fields.

### 2.9 Colony formation assay

200 untreated cells were seeded in six-well plate and cultivated for 2 weeks. Afterwards, cells were fixed with methanol for 15 minutes and stained with 0.4% crystal violet for 30 minutes at room temperature, and the colonies were analyzed.

### 2.10 EDU proliferation assay

20,000 untreated cells were seeded in 96-well plate and cultivated for 24 hours. EDU proliferation assay was performed following the instruction of BeyoClick™ EdU Cell Proliferation Kit with DAB (Beyotime, Nanjing, China), and the cells were then observed using fluorescent microscope.

### 2.11 Immunoassay analysis

Utilizing heatmaps for immune infiltration and correlation maps, the degree of tumor invasion was compared to the model. CIBERSORT and XCELL methods were mainly refered (28, 29). A list of genes related to immune checkpoints was discovered (30–33). The boxplots depicted the results of the analyses.

### 2.12 Drug sensitivity analysis

We received expression matrices and medication processing data from the Cancer Genome Project. The drugs associated with the prognosis model were derived using the “pRROpheticPredict” tool (p<0.001) (34).

## 3 Results

### 3.1 Data processing

After screening, 365 patients with HCC were included in the study. Using Pearson correlation analysis, we obtained 415 m6A-associated lncRNAs and 488 NETs-related lncRNAs. Then, we obtained a total of 209 MNlncRNAs for subsequent analysis after taking the intersection of the two sets of lncRNAs.

### 3.2 Prognostic model construction

**Figure 1:**
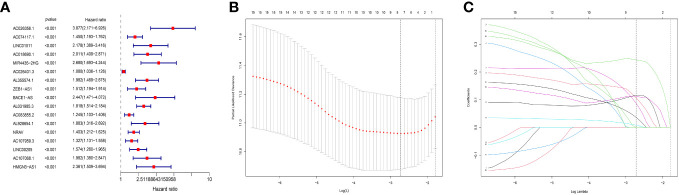
Prognostic model construction. **(A)** Univariate COX analysis. 17 predictive N6-methyladenosine- and neutrophil extracellular traps- related long non-coding RNA (MNlncRNAs) were chosen. MNlncRNAs in red are classified as high risk. **(B, C)** The least absolute selection operator (LASSO) regression analysis. LASSO regression analysis was used to select additional predictive MNlncRNAs.

First, we performed a different analysis and 148 MNlncRNAs with differences in the normal and tumor groups were screened out. After matching the MNlncRNAs expression data and clinical data, we performed the univariate COX analysis and selected 17 MNlncRNAs (**Figure 1A**). Then through Lasso regression analysis, we selected out 7 MNlncRNAs and built the prognostic model (**Figures 1B, C**). The model was calculated as follows: risk score = AC074117.1* 0.10679103 + AC026401.3* 0.00817401 + AL355574.1* 0.08725795 + ZEB1.AS1* 0.11541016 + AL031985.3* 0.24377726 + NRAV* 0.11141409 + AC107959.3* 0.03665612.

**Figure 2:**
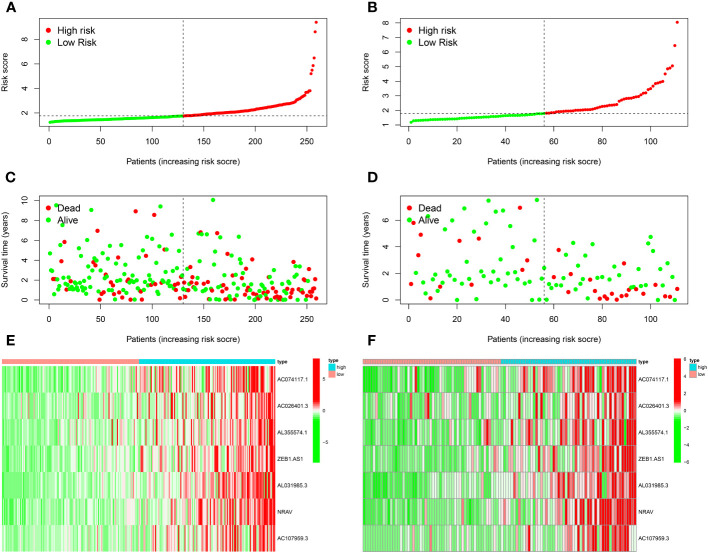
Prognostic model evaluation. **(A, B)** The risk score of training **(A)** and test **(B)** cohorts. **(C, D)** The correlation between survival status and riskscore. **(E, F)** Expression heatmap of 7 model MNlncRNAs.

**Figure 3:**
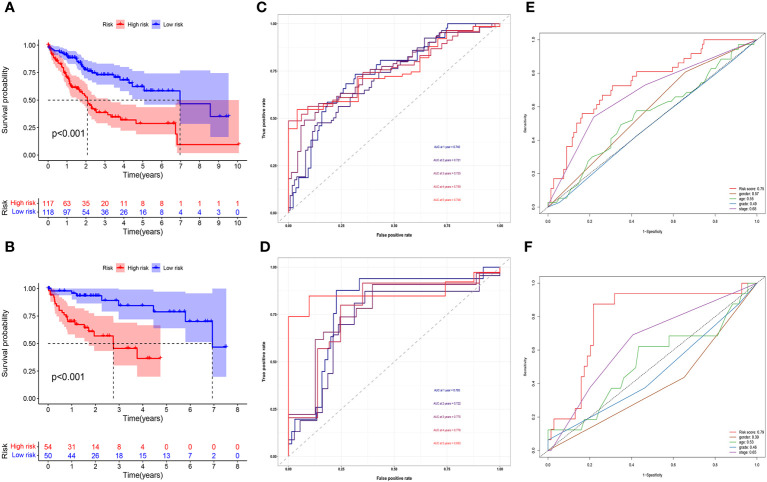
Prognostic model evaluation. **(A, B)** High-risk patients did poorly in both the training **(A)** and test cohorts **(B)**. **(C, D)** The AUC in both cohorts was essentially in the 0.7–0.9 range. **(E, F)** Risk score had the highest predictive accuracy compared with clinical characteristics in both cohorts.

**Figures 2A, B** demonstrated how we classified HCC samples as high- or low-risk. The risk score increased the percentage of patients who died (**Figures 2C, D**). Besides, among those at high risk, all the model MNlncRNAs were substantially expressed in both cohorts (**Figures 2E, F**). The results of the survival research showed that the high-risk group’s outcome was much poorer (**Figures 3A, B**). For the training cohort, the AUCs were 0.740, 0.755, and 0.749 at 1, 3, and 5 years, respectively (**Figure 3C**). For the test cohort, the AUCs were 0.795, 0.775, and 0.863 at 1, 3, and 5 years, respectively (**Figure 3D**). Finally, to further evaluate the prognostic value of the model, we compared it with clinical characteristics (TNM stage, age, grade and gender) and found that the model had the highest predictive accuracy (**Figures 3E, F**).

### 3.3 Functional analysis

**Figure 4:**
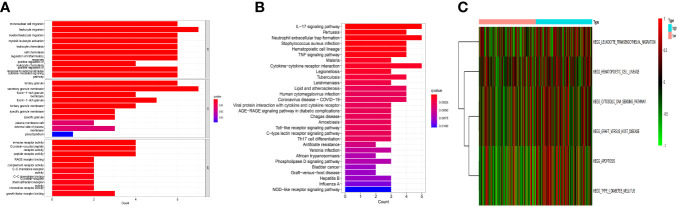
Functional analysis. **(A)** The Gene Ontology (GO) analysis. These genes functions were stimulation of mononuclear migration and leukocyte migration in BP, tertiary granule and secretory granule membrance in CC, and immune receptor activity and receptor activity in MF. **(B)** The Kyoto Encyclopedia of Genes and Genomes (KEGG) analysis. They were mainly involved in cytokine−cytokine receptor interaction, neutrophil extracellular trap formation, and immune-related signaling pathways. **(C)** The gene set variation analysis (GSVA). They were mainly involved in leukocyte migration, signal transmission, and apoptosis.

The key roles of these genes, as determined by the results of the GO enrichment study, were stimulation of mononuclear migration and leukocyte migration in BP, tertiary granule and secretory granule membrance in CC, and immune receptor activity and receptor activity in MF (**Figure 4A**). Besides, they mostly contributed to neutrophil extracellular trap formation, immune-related signaling pathways, and cytokine−cytokine receptor interaction by the KEGG analysis (**Figure 4B**). The GSVA analysis showed that they also contributed to leukocyte migration, signal transmission, and apoptosis (**Figure 4C**).

### 3.4 Model validation *in vitro* and *vivo*

**Figure 5:**
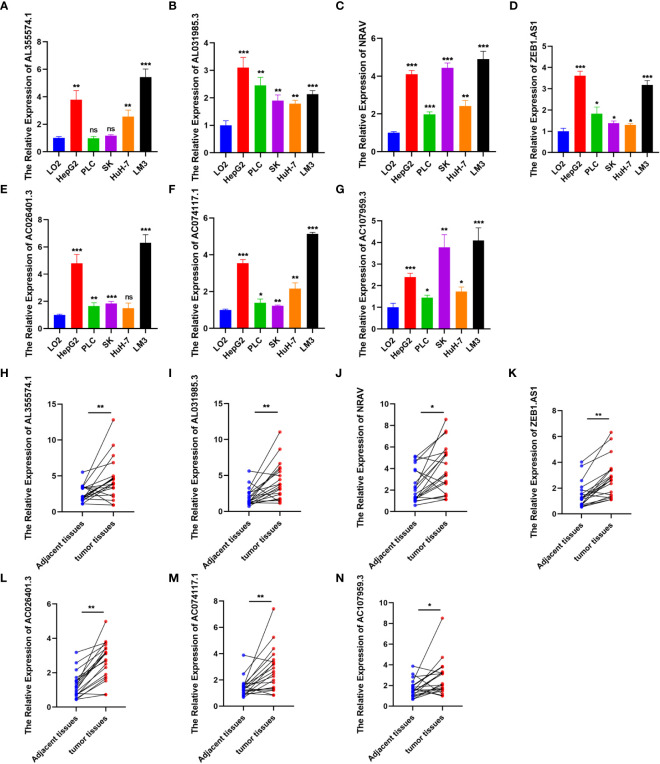
Expression level analysis. **(A–G)** The expression of AL355574.1, AL031985.3, NRAV, ZEB1.AS1, AC026401.3, AC074117.1 and AC107959.3 in normal liver cell and HCC cells were detected by RT-qPCR. **(H–N)** Paired comparison of the differential expression of the 7 lncRNAs between 20 HCC tissues and adjacent tissues. *p<0.05; **p<0.01; ***p<0.001; ns, not significant.

**Figure 6:**
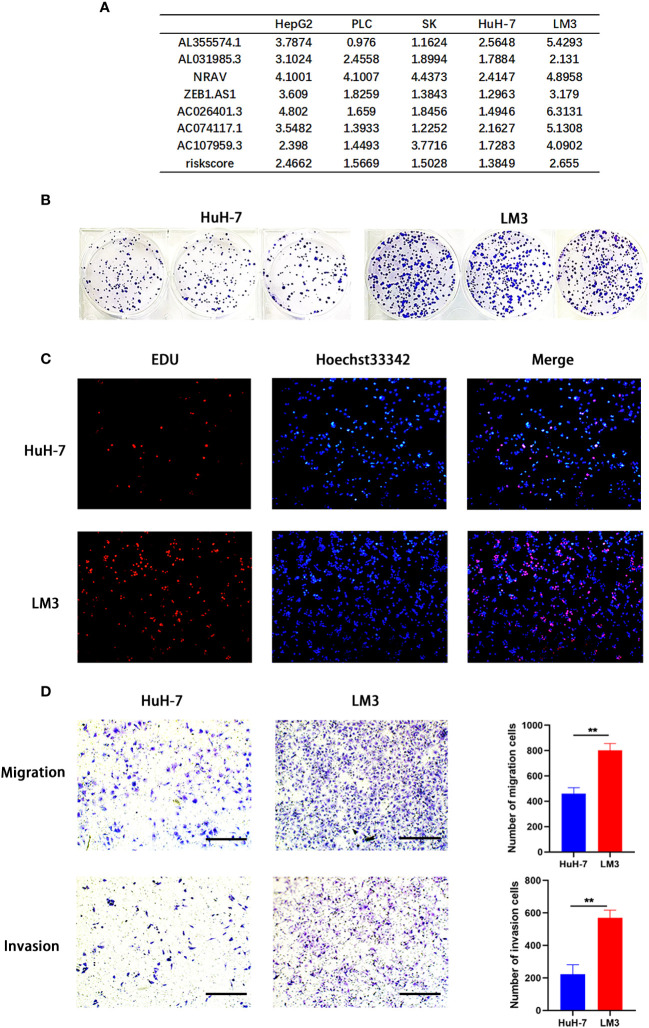
Model validation *in vitro*. **(A)** Application of risk model to hepatocellular carcinoma (HCC) cell lines. **(B–D)** Colony formation assay, EDU assay, and transwell migration and invasion assay (Scale bar: 100mm) showing that LM3 had relatively higher malignancy than HuH-7. **p<0.01.

To further verify the reliability of the risk model, we detected the expression of the 7 genes by using qRT-PCR. The results showed significantly increased expression of these genes in HCC cells (PLC, SK, LM3, HepG2 and HuH-7) (**Figures 5A–G**) and tumor tissues from 20 patients diagnosed with hepatocellular carcinoma (**Figures 5H–N**), consistent with these of the bioinformatics analysis. Afterwards, we calculated the risk score of hepatocellular carcinoma cell lines according above calculation method. As shown in **Figure 6A**, the risk scores of HepG2 (2.4662) and LM3 (2.655) were relatively close. Similarly, the risk scores of PLC (1.5669), SK (1.5028) and HuH-7 (1.3849) were also relatively close. Based on our model, we believe that cells with similar risk scores have little difference in biological characteristics, so we selected cells with the highest and lowest risk scores for functional experiments. Subsequently, we further compared the ability of proliferation and metastasis of LM3 and HuH-7. Colony formation and EDU proliferation assay indicted that LM3 cells proliferation rate was significantly higher than HuH-7 cells (**Figures 6B, C**). Meanwhile transwell migration and invasion assay was performed, which showed that LM3 cells had higher metastatic potential (**Figure 6D**). The results confirmed that LM3 had relatively higher malignancy than other cell lines, suggesting that patients with higher risk score had worse prognosis.

### 3.5 Immunoassay analysis

**Figure 7:**
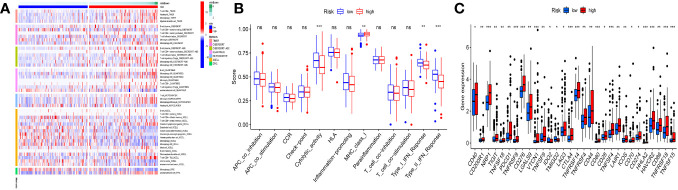
Immunoassay analysis. **(A)** Significant correlations existed between risk scores and B cells, T cells, monocyte cells, and macrophage cells. **(B)** Low-risk populations have more active immune systems. **(C)** Most immune checkpoint genes had increased expression in the high-risk group. *p<0.05; **p<0.01; ***p<0.001; ns, not significant.

The immune microenvironment had a significant impact on tumor development. B cell, monocyte, T cell, and macrophage immunocorrelation research revealed a high correlation between risk scores and each of these cell types (**Figure 7A**). In the low-risk group, the Cytolytic_activity, the Type-II-IFN_Reponse, and Type-I-IFN-Response were more activate; In the high-risk group, the MHC_class-I were more activated (**Figure 7B**). Significant differences in immunological checkpoint gene expression were found between the two groups, as demonstrated by **Figure 7C**. In the low-risk group, the immune system was more active, indicating that immunotherapy could be beneficial for these individuals. Furthermore, the high-risk group had higher levels of the vast majority of immune checkpoint genes, which raises the possibility that the immunological milieu may differ between the two groups.

### 3.6 Drug sensitivity analysis

**Figure 8:**
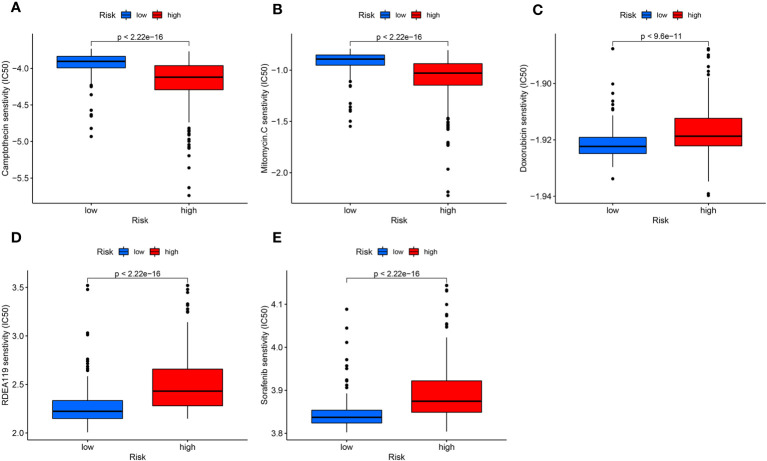
Drug sensitivity analysis. **(A, B)** Camptothecin **(A)** and Mitomycin.C **(B)** sensitivity was higher in the high-risk group. **(C-E)** Doxorubicin **(C)**, Refametinib **(D)**, and Sorafenib **(E)** were more toxic to the low-risk group.

We carry out drug susceptibility testing to find effective medications for targeted therapy. After analysis, in the high-risk group, Camptothecin and Mitomycin.C, were more sensitive (**Figures 8A, B**). Doxorubicin, Refametinib, and Sorafenib were more sensitive in the low-risk group (**Figures 8C–E**).

## 4 Discussion

We used in-depth bioinformatics analysis to investigate the significance of lncRNAs associated with m6A and NETs in HCC in this work. Based on the TCGA database, we constructed a prognostic model based on MNlncRNAs expression, which can accurately predict their prognosis and stratify HCC patients. Additionally, our research revealed substantial variations in the function of MNlncRNAs in the immunological milieu of HCC, which may provide HCC patients a new therapy predictor. By finding more sensitive drugs, drug sensitivity analysis helps stratify the treatment of HCC.

LncRNAs are connected to carcinogenesis and metastasis through aberrant expression, and they are a hub in the area of cancer diagnosis, therapy, and prognosis evaluation (35, 36). LncRNA can promote or inhibit tumor progression. There have been reports that certain lncRNAs might serve as biomarkers for HCC patients with high sensitivity or be involved in important pathways of tumor regulation (37). It was shown that lncRNA-PDPK2P promotes the progression of HCC by interacting with PDK1, thereby affecting the value-added and migration of HCC (38). Prognostic markers of lncRNAs can be used to differentiate immunotherapy responses in cancer patients (39). Although lncRNA-based prognostic models are beneficial for the prognostic assessment of HCC patients, they have the disadvantage of many variables and lack accuracy (37). To increase the precision of lncRNA prognostic models, we tried to combine m6A and NETs inspired by the study of Huang et al (24). Thus, we first identified a set of specific lncRNAs linked to m6A and NETs genes in our study, which were used to construct MNlncRNAs markers. After combining the two prognostic biomarkers, we constructed a prognostic model with superior accuracy, with an AUC value for survival prediction essentially greater than 0.7. In conclusion, the construction of MNlncRNAs-based prognostic models is a great innovation that has superior predictive power than common clinical prognostic models.

According to preliminary results, the prognostic model’s model lncRNAs have been linked to the illness’s onset and progression. AC074117.1 was regulated by super-enhancers, and its silencing significantly slowed the growth of lung cancer cells (40). Yao et al. constructed a 4-lncRNA model to estimate the prognosis of individuals with breast cancer in which AC026401.3 played a key role (41). In the 7-lncRNA prognostic model developed by Yuan et al., AL355574.1 was a key component to assess the prognosis of gastric cancer (42). It has been demonstrated that increased ZEB1.AS1 expression is linked to tumor development and metastasis (43). The study by Li et al. screened 15 lncRNAs to estimate the prognosis of individuals with colorectal cancer, and AL031985.3 played a key role in this (44). NRAV promoted pancreatic cancer progression by targeting miR-299-3p (45). Huang et al. constructed an 8-lncRNA model to estimate the prognosis of individuals with HCC in which AC107959.3 played an important role (46). These 7 lncRNAs were included in the prognostic model used in this work, which can help us better understand tumor development.

Death in patients with tumors is more often attributed to metastases than to the primary tumor. The majority of patients die as a result of organ failure, cancer-associated thrombosis, or other problems connected to tumor spread (47). There is growing evidence that heterogeneity remains between tumors in the same tissue. The energy and nourishment that the tumor microenvironment provides to sustain tumor development and spread is essential for tumor growth (48). Neutrophils, the most common cells in the human immune system, may serve as a bridge between the tumor parenchyma and the immunological microenvironment. Neutrophil extracellular traps are an important mechanism in innate immunity that is implicated in cancer development and has recently emerged as a hotspot (49). NETs are expected to play an important function in the tumor immune microenvironment. NETs are a distinct kind of neutrophil that protects tumor cells from immune assault, activates dormant tumor cells, and promotes tumor invasion and metastasis (50). The colocalization between tumor cells and NETs is closely linked, which would also promote tumor progression (50). Recent studies have provided a preliminary exploration of the mechanisms by which NETs promote tumor metastasis. NETs can upregulate the TLR9 pathway to promote the progression of diffuse large B lymphoma (51). CTSC enzymes released by breast cancer cells can control NET development, thus promoting lung metastasis of breast cancer (52). The tumor microenvironment of HCC can promote early metastasis of HCC by facilitating tumor cell invasion and migration (53). In recent years, advances in epigenetic techniques provide researchers with new insights into tumor research (54). An increasing number of studies have focused on tumorigenic progression and m6A modifications of lncRNAs in innate immunity (55). The most prevalent RNA modification, M6A, is crucial for many cellular processes and biological functions (56, 57). Factors that affect m6A alteration are linked to both specific malignancies and abnormal immune regulation (58, 59). The tumor microenvironment is highly correlated with M6A alterations (60). Additionally, tumor immunotherapy benefits from aspects of tumor microenvironment cell invasion mediated by m6A regulator (61). Our findings revealed a set of lncRNA signatures associated with m6A and NETs that helped us understand the progression of HCC. The risk score classified all HCC patients into two groups. The majority of cancer progression occurred in high-risk individuals, whereas other patients lived longer.

HCC is a disease that is fueled by inflammation, and a sizable portion of HCC patients show indicators of the inflammatory response (62). Stromal and tumor cells can produce immunosuppression associated with chronic inflammatory factors (63). Cells of bone marrow origin, including tumor-associated neutrophils (TANs), promote tumor progression (64). It has been demonstrated that TANs encourage HCC development, progression, and sorafenib resistance by enlisting macrophages and T cells into the tumor’s microenvironment (65). TAN-induced HCC stem-like cells are active in signaling, CXCL5 secretion, and recruitment of more TAN infiltration (66). Also, a poor prognosis is linked to the presence of neutrophils in the tumor microenvironment (67). Importantly, the knockdown of CXCR2, a key chemotactic receptor for neutrophils, to inhibit immune infiltration of neutrophils can lead to a T cell-dependent suppression of tumor growth (68). Therefore, understanding the immune infiltration of neutrophils is crucial to the diagnosis and management of HCC. Our study stratified patients according to modeled risk scores. The high-risk patients had more immune infiltrating cells, and the majority of immunological checkpoint-related genes have increased expression, indicating that immunotherapy may help the high-risk patients more. For patients in the two groups, we screened for drugs that are sensitive to their treatment, which helped to individualize the treatment for patients.

However, our study possesses certain limitations. The first limitation pertains to the inadequate elucidation of the mechanisms underlying the functionality of these m6A and neutrophil extracellular traps-related lncRNAs. Their role in shaping the tumor microenvironment and tumor growth and progression remains ambiguous, necessitating further investigation. Furthermore, this model is constructed based on the TCGA public database and lacks validation with a larger sample size. Despite these limitations, it is the initial model created incorporating lncRNAs associated with m6A and NETs. It provides information for studying the metabolism of HCC tumors and aids in the treatment of HCC patients.

## 5 Conclusion

Based on m6A- and NETs-related lncRNAs, a prognostic model of HCC was established. This model can accurately predict the immune microenvironment and prognosis of HCC patients. In addition, our findings could result in new approaches.

## Data availability statement

The original contributions presented in the study are included in the article/**Supplementary Material**. Further inquiries can be directed to the corresponding authors.

## Ethics statement

The studies involving humans were approved by The Second Affiliated Hospital of Nanjing Medical University. The studies were conducted in accordance with the local legislation and institutional requirements. The participants provided their written informed consent to participate in this study.

## Author contributions

TZ, WW, and XG designed the study. XG and WB were involved in database search and statistical analyses. TZ and NL were involved in the writing of the manuscript. NL and JZ were responsible for the submission of the final version of the paper. All authors contributed to the article and approved the submitted version.
