# Supplementary material for: Construction of an m6A- and neutrophil extracellular traps-related lncRNA model to predict hepatocellular carcinoma prognosis and immune landscape
Source: Front Immunol. 2023 Oct 5;14:1231543. doi: 10.3389/fimmu.2023.1231543 (PMC10585104; doi:10.3389/fimmu.2023.1231543)
Supplement: Supplementary file 1 [file DataSheet_1.pdf]

Table S1. Premiers for RT-qPCR.

ID	**Sequence (5' – 3')**
AC074117.1-F	TAGGGTACACAAGGTGATCTGC
AC074117.1-R	CCCCACCTCCCCCAATACTA
AL355574.1-F	GGAGGGCAGAGAGCAACGTA
AL355574.1-R	CGCCTCTACAGACAGCACTC
ZEB1-AS1-F	GAACCGGGATGGGAAGTGAC
ZEB1-AS1-R	GCAAGCGGAACTTCTAGCCT
AL031985.3-F	AAATCCCATACCCCTTTCACC
AL031985.3-R	TTTACTGAGTCCCTTCTGCGTG
NRAV-F	GGAGTTGATGCCTCCGAACA
NRAV-R	ATGACCGGAGCTGAAAGGTG
AC107959.3-F	AATCAGCTGTGCTCTCTCCC
AC107959.3-R	CAGGAGACAAAGGTGTCCGT
AC026401.3-F	TCTCCACGCATCTGCACAAT
AC026401.3-R	GCTGTTACGCCACGGTTTTT
GAPDH-F	CATGAGAAGTATGACAACAGCCT
GAPDH-R	AGTCCTTCCACGATACCAAAGT
